# Crystal structure and Hirshfeld surface analysis of (2*Z*)-*N*,*N*-dimethyl-2-(penta­fluoro­phen­yl)-2-(2-phenyl­hydrazin-1-yl­idene)acetamide

**DOI:** 10.1107/S2056989021007349

**Published:** 2021-07-23

**Authors:** Zeliha Atioğlu, Mehmet Akkurt, Namiq Q. Shikhaliyev, Ulviyya F. Askerova, Aytan A. Niyazova, Sixberth Mlowe

**Affiliations:** a Department of Aircraft Electrics and Electronics, School of Applied Sciences, Cappadocia University, Mustafapaşa, 50420 Ürgüp, Nevşehir, Turkey; b Department of Physics, Faculty of Sciences, Erciyes University, 38039 Kayseri, Turkey; c Organic Chemistry Department, Baku State University, Z. Xalilov str. 23, Az, 1148 Baku, Azerbaijan; d Azerbaijan State University of Economics (UNEC), M. Mukhtarov str.194, Baku, Azerbaijan; e University of Dar es Salaam, Dar es Salaam University College of Education, Department of Chemistry, PO Box 2329, Dar es Salaam, Tanzania

**Keywords:** crystal structure, fluorine, hydrogen bonds, π–π stacking inter­actions, SQUEEZE, Hirshfeld surface analysis

## Abstract

In the title compound, C_16_H_12_F_5_N_3_O, the dihedral angle between the aromatic rings is 31.84 (8)°. In the crystal, the mol­ecules are linked into dimers possessing crystallographic twofold symmetry by pairwise N—H⋯O hydrogen bonds and weak C—H⋯O hydrogen bonds and aromatic π–π stacking inter­actions link the dimers into a three-dimensional network. A Hirshfeld surface analysis indicates that the most important contributions to the crystal packing are from F⋯H/H⋯F (41.1%), H⋯H (21.8%), C⋯H/H⋯C (9.7%) C⋯C (7.1%) and O⋯H/H⋯O (7.1%) contacts. The contribution of some disordered solvent to the scattering was removed using the SQUEEZE routine [Spek (2015 ▸). *Acta Cryst*. C**71**, 9–18] in *PLATON*. The solvent contribution was not included in the reported mol­ecular weight and density.

## Abstract 2

The dihedral angle between the aromatic rings in the title compound is 31.84 (8)°; N—H⋯O and C—H⋯O hydrogen bonds and π–π stacking inter­actions connect mol­ecules in the crystal, producing a three-dimensional network.

## Chemical context   

Aryl­hydrazones containing a (Ph,*R*)C=N—NH*R* grouping possess controllable *E*/*Z* isomerization around the C=N double bond, which makes them good candidates for the construction of functional materials (Ma *et al.*, 2021 ▸). Control of the supra­molecular chemistry of hydrazone ligands and the corresponding complexes may afford multi-dimensional synthons or metallo-organic tectons (Kopylovich *et al.*, 2011 ▸; Gurbanov *et al.*, 2020*a* ▸). The functionalization of aryl­hydrazone ligands with groups such as –SO_3_H, –COOH, –F, –Cl, *etc*., can improve the catalytic or biological activity of the corresponding coordination compounds (*e.g.*, Shikhaliyev *et al.*, 2019 ▸; Gurbanov *et al.*, 2020*b* ▸). As part of our ongoing work in this area, we have synthesized the title fluorinated aryl­hydrazone compound, C_16_H_12_F_5_N_3_O, and determined its crystal structure and analysed its Hirshfeld surface. 
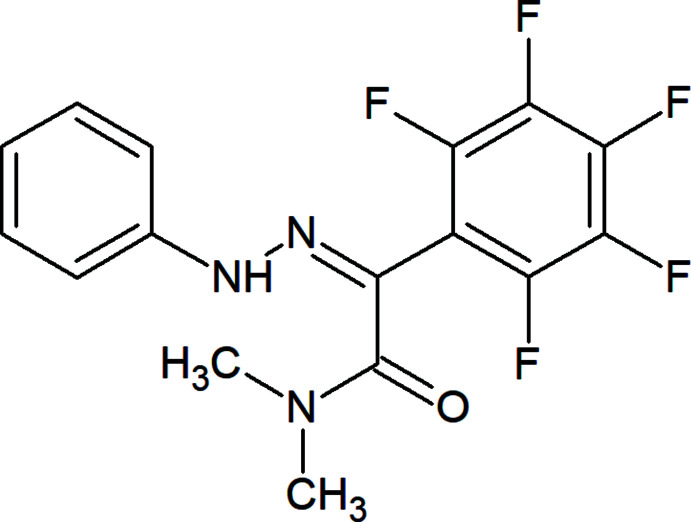


## Structural commentary   

**Figure 1:**
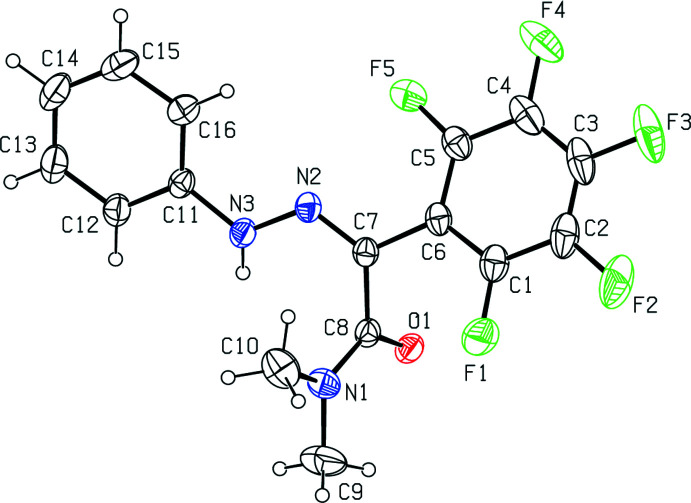
The title mol­ecule showing 30% probability displacement ellipsoids.

The title mol­ecule (Fig. 1 ▸) crystallizes in the monoclinic space group *C*2/*c* with *Z* = 8 and has an *E* conformation with an azomethine N2=C7 double bond length of 1.2880 (16) Å. The backbone of the mol­ecule is non-planar with a dihedral angle of 31.84 (8)° between the C1–C6 penta­flouro­benzene and C11–C16 benzene rings and the acetamide group lies almost perpendicular. The C5—C6—C7—N2, C6—C7—N2—N3, C7—N2—N3—C11, N2—N3—C11—C16 and C6—C7—C8—N1 torsion angles are −28.19 (17), 174.02 (10), −176.33 (11), 5.90 (18) and 122.80 (12)°, respectively.

## Supra­molecular features   

**Figure 2:**
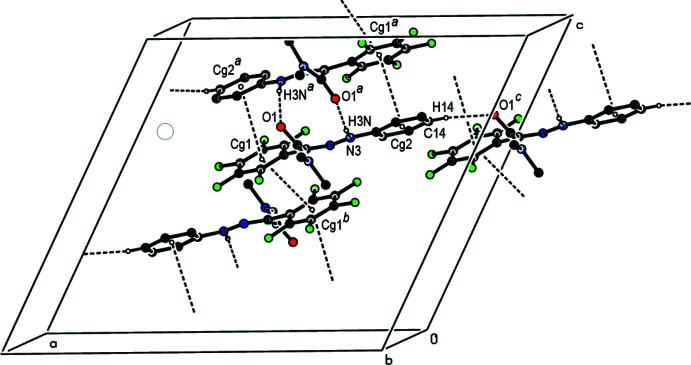
View oblique to [010] of the inter­molecular N—H⋯O, C—H⋯O and π–π stacking inter­actions of the title compound.

In the crystal, the mol­ecules are linked by pairwise N—H⋯O hydrogen bonds (Table 1 ▸), generating dimers featuring an 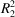 (12) loop with crystallographic twofold symmetry. The dimers are linked by C—H⋯O hydrogen bonds and aromatic π–π stacking inter­actions [*Cg*1⋯*Cg*1^*b*^ = 3.7137 (10) Å, slippage = 1.158 Å, *Cg*1⋯*Cg*2^*b*^ = 3.7015 (9) Å, slippage = 1.407 Å, and *Cg*1⋯*Cg*2^*a*^ = 3.7016 (9) Å, slippage = 1.148 Å; where *Cg*1 and *Cg*2 are the centroids of the C1–C6 and C11–C16 rings, respectively; symmetry codes: (*a*) 1 − *x*, *y*, 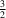 − *z*; (*b*) 1 − *x*, 1 − *y*, 1 − *z*]. Together, these generate a three-dimensional network (Fig. 2 ▸).

## Hirshfeld surface analysis   

**Figure 3:**
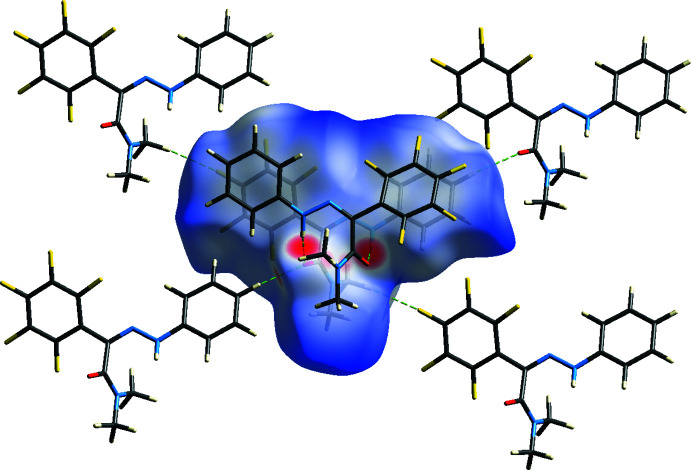
View of the three-dimensional Hirshfeld surface of the title compound plotted over *d*_norm_ in the range −0.52 to 2.23 a.u.

*Crystal Explorer 17.5* was used to calculate the Hirshfeld surfaces and two-dimensional fingerprint plots (Turner *et al.*, 2017 ▸). The three-dimensional Hirshfeld surface mapped over *d*_norm_ in the range −0.52 to 2.23 a.u. is shown in Fig. 3 ▸: the H9*C*⋯F1, H16⋯F2, F3⋯H10*C*, H3*N*⋯O1, N3—H3*N*⋯O1 and C14—H14⋯O1 inter­actions, which play a key role in the mol­ecular packing, can be correlated with the bright-red patches near F1, F2, F3 and O1 and hydrogen atoms H3*N* and H14, which highlight their functions as donors and/or acceptors. This may be compared to the Hirshfeld surface mapped over electrostatic potential (Spackman *et al.*, 2008 ▸) depicted in the supporting information corresponding to positive electrostatic potential (hydrogen-bond donors) in blue and negative electrostatic potential is indicated in red (hydrogen-bond acceptors).

**Figure 4:**
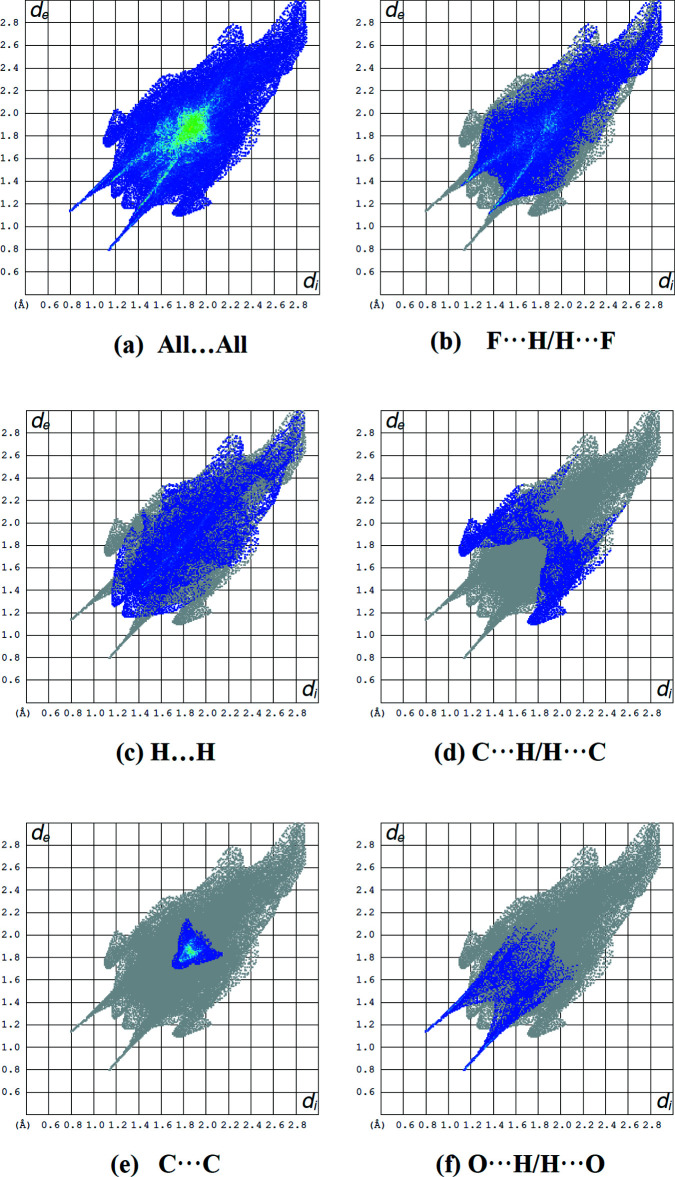
Two-dimensional fingerprint plots for the title compound showing (*a*) all inter­actions, and delineated into (*b*) F⋯H/H⋯F, (*c*) H⋯H, (*d*) C⋯H/H⋯C, (*e*) C⋯C and (*f*) O⋯H/H⋯O inter­actions. The *d*_i_ and *d*_e_ values are the closest inter­nal and external distances (in Å) from given points on the Hirshfeld surface.

The overall two-dimensional fingerprint map for the title compound is shown in Fig. 4 ▸ *a*. The percentage contributions to the Hirshfeld surfaces from various inter­atomic contacts (Table 2 ▸) are F⋯H/H⋯F (41.1%; Fig. 4 ▸ *b*), H⋯H (21.8%; Fig. 4 ▸ *c*), C⋯H/H⋯C (9.7%; Fig. 4 ▸ *d*) C⋯C (7.1%; Fig. 4 ▸ *e*) and O⋯H/H⋯O (7.1%; Fig. 4 ▸ *f*). Other contact types including N⋯H/H⋯N, N⋯C/C⋯N and N⋯N contacts account for less than 5.4% of the Hirshfeld surface mapping and presumably have minimal directional impact on the packing.

## Database survey   

The five related compounds in the Cambridge Structural Database (CSD Version 5.42, update 1, Feb 2021; Groom *et al.*, 2016 ▸) with a (1*E*)-1-benzyl­idene-2-phenyl­hydrazine skeleton are (*E*)-3-chloro-*N*′-(2-fluoro­benzyl­idene)thio­phene-2-carbohydrazide (refcode SOJQAL: Sultan *et al.*, 2014 ▸), *N*′-[1-(2-fluoro­phen­yl)ethyl­idene]isonicotinohydrazide (HIX­RAJ: Sreeja *et al.*, 2014*a* ▸), (1*E*,2*E*)-bis­[(thio­phen-2-yl)meth­yl­idene]hydrazine (MIHROK03: Geiger *et al.*, 2013 ▸), *N*′-[1-(2-fluoro­phen­yl)ethyl­idene]nicotinohydrazide (ZISSAX: Sreeja *et al.*, 2014*b* ▸) and 4-[1-(4-chloro­phen­yl)-3-oxo­butyl­amino]­benzoic acid (TINWIX: Narayana *et al.*, 2007 ▸).

The hydrazide derivative SOJQAL adopts an *E* conformation with an azomethine N=C double bond length of 1.272 (2) Å. The mol­ecular skeleton is approximately planar, the terminal five- and six-membered rings forming a dihedral angle of 5.47 (9)°. In the crystal, mol­ecules are linked by N—H⋯O and C—H⋯O hydrogen bonds into zigzag chains propagating in [100].

The mol­ecule of HIXRAJ adopts an *E* conformation with respect to the azomethine bond. The pyridyl and fluoro­benzene rings make dihedral angles of 38.58 (6) and 41.61 (5)° respectively with the central C(=O)N_2_CC unit, resulting in a non-planar mol­ecule. The inter­molecular inter­actions comprise two classical N—H⋯O and N—H⋯N hydrogen bonds and four non-classical C—H⋯O and C—H⋯F hydrogen bonds. These inter­actions are augmented by a weak π–π inter­action between the benzene and pyridyl rings of neighbouring mol­ecules, with a centroid–centroid distance of 3.9226 (10) Å. This leads to a three-dimensional supra­molecular assembly in the crystal.

The asymmetric unit of MIHROK03 comprises two independent half-mol­ecules, each residing on a centre of symmetry. The two mol­ecules are essentially planar. In the crystal, weak C—H⋯π inter­actions join the two symmetry-independent mol­ecules into inter­linked chains parallel to [011].

The mol­ecule of ZISSAX adopts an *E* conformation with respect to the azomethine double bond whereas the N and methyl C atoms are in a *Z* conformation with respect to the same bond. The ketonic O and azomethine N atoms are *cis* to each other. The non-planar mol­ecule [the dihedral angle between the benzene rings is 7.44 (11)°] exists in an amido form with a C=O bond length of 1.221 (2) Å. In the crystal, a bifurcated N—H⋯(O,N) hydrogen bond is formed between the amide H atom and the keto O and imine N atoms of an adjacent mol­ecule, leading to the formation of chains propagating along the *b*-axis direction.

In TINWIX, the aromatic rings are almost perpendicular, making a dihedral angle of 89.26 (5)°. The carboxyl group is coplanar with the aromatic ring to which it is attached [dihedral angle = 1.70 (17)°]. The packing involves inversion-symmetric dimers bridged *via* hydrogen bonding of the carboxyl groups. In addition, there is an N—H⋯O hydrogen bond between the amino group and the carbonyl O atom.

## Synthesis and crystallization   

A 20 ml screw-neck vial was charged with DMSO (10 ml), (*E*)-1-[(perfluoro­phen­yl)methyl­ene]-2-phenyl­hydrazine (286 mg, 1.00 mmol), tetra­methyl­ethylenedi­amine (TMEDA) (295 mg, 2.50 mmol), CuCl (2 mg, 0.02 mmol) and CCl_4_ (20 mmol, 10 equiv). After 1–3 h (until TLC analysis showed complete consumption of the corresponding Schiff base), the reaction mixture was poured into a 0.01 *M* solution of HCl (100 ml, pH = 2–3), and extracted with di­chloro­methane (3 × 20 ml). The combined organic phase was washed with water (3 × 50 ml), brine (30 ml), dried over anhydrous Na_2_SO_4_ and concentrated *in vacuo* using a rotary evaporator. The residue was purified by column chromatography on silica gel using appropriate mixtures of hexane and di­chloro­methane (3/1–1/1). Colourless prisms of the title compound suitable for X-ray analysis were obtained by slow evaporation of a di­chloro­methane solution (69%); m.p. 405 K. Analysis calculated for C_16_H_12_F_5_N_3_O: C 53.79, H 3.39, N 11.76; found: C 53.73, H 3.36, N 11.71%. ^1^H NMR (300MHz, CDCl_3_) *δ* 3.04 (6H, NMe_2_), 6.50–7.33 (5H, Ar). ^13^C NMR (75MHz, CDCl_3_) *δ* 33.58, 108.97, 116.87, 120.75, 124.11, 124.76, 140.95, 146.33, 149.87, 150.91, 155.21. ESI–MS: *m*/*z*: 358.24 [*M* + H]^+^.

## Refinement   

Crystal data, data collection and structure refinement details are summarized in Table 3 ▸. The H atom of the NH group was found from a difference-Fourier map and refined freely. All H atoms bonded to C atoms were positioned geometrically and treated as riding atoms, with C—H = 0.93 or 0.96 Å, and with *U*_iso_(H) = 1.2 or 1.5*U*_eq_ (C). The residual electron density was difficult to model and therefore the SQUEEZE routine (Spek, 2015 ▸) in *PLATON* (Spek, 2020 ▸) was used to remove the contribution of the electron density in the solvent region from the intensity data and the solvent-free model was employed for the final refinement. The solvent formula mass and unit-cell characteristics were not taken into account during refinement. The cavity of volume *ca* 255.0 Å^3^ (*ca* 7.6% of the unit-cell volume) contains approximately three electrons.

## Supplementary Material

Crystal structure: contains datablock(s) I, global. DOI: 10.1107/S2056989021007349/hb7979sup1.cif

Structure factors: contains datablock(s) I. DOI: 10.1107/S2056989021007349/hb7979Isup2.hkl

Electrostatic potential map. DOI: 10.1107/S2056989021007349/hb7979sup3.docx

Supporting information file. DOI: 10.1107/S2056989021007349/hb7979Isup4.cml

CCDC reference: 1878189

Additional supporting information:  crystallographic information; 3D view; checkCIF report

## Figures and Tables

**Table 1:** Hydrogen-bond geometry (Å, °) *Cg*2 is the centroid of the C11–C16 ring.

*D*—H⋯*A*	*D*—H	H⋯*A*	*D*⋯*A*	*D*—H⋯*A*
N3—H3*N*⋯O1^i^	0.87 (1)	2.05 (2)	2.8658 (15)	154 (1)
C14—H14⋯O1^ii^	0.93	2.45	3.377 (2)	172
C10—H10*B*⋯*Cg*2^iii^	0.96	2.77	3.4685 (19)	130

Symmetry codes: (i) -x+1, y, -z+{\script{3\over 2}}; (ii) x-{\script{1\over 2}}, y-{\script{1\over 2}}, z; (iii) -x+{\script{1\over 2}}, -y+{\script{3\over 2}}, -z+1.

**Table 2:** Percentage contributions of inter­atomic contacts to the Hirshfeld surface for the title compound

Contact	Percentage contribution
F⋯H/H⋯F	41.1
H⋯H	21.8
C⋯H/H⋯C	9.7
C⋯C	7.1
O⋯H/H⋯O	7.1
F⋯F	5.4
F⋯C/C⋯F	4.1
F⋯N/N⋯F	1.5
N⋯C/C⋯N	1.1
O⋯O	0.3
N⋯N	0.2
O⋯C/C⋯O	0.2
N⋯H/H⋯N	0.1

**Table 3:** Experimental details

Crystal data	
Chemical formula	C_16_H_12_F_5_N_3_O
*M*_r_	357.29
Crystal system, space group	Monoclinic, *C*2/*c*
Temperature (K)	296
*a*, *b*, *c* (Å)	19.0048 (6), 11.5216 (4), 17.2227 (6)
β (°)	116.526 (1)
*V* (Å^3^)	3374.2 (2)
*Z*	8
Radiation type	Mo *K*α
μ (mm^−1^)	0.13
Crystal size (mm)	0.86 × 0.76 × 0.32

Data collection	
Diffractometer	Bruker APEXII CCD
Absorption correction	Multi-scan (*SADABS*; Krause *et al.*, 2015 ▸)
*T*_min_, *T*_max_	0.666, 0.746
No. of measured, independent and observed [*I* > 2σ(*I*)] reflections	20072, 3619, 3075
*R*_int_	0.025
(sin θ/λ)_max_ (Å^−1^)	0.639

Refinement	
*R*[*F* ^2^ > 2σ(*F* ^2^)], *wR*(*F* ^2^), *S*	0.044, 0.129, 1.08
No. of reflections	3619
No. of parameters	232
No. of restraints	1
H-atom treatment	H atoms treated by a mixture of independent and constrained refinement
Δρ_max_, Δρ_min_ (e Å^−3^)	0.27, −0.16

Computer programs: *APEX3* and *SAINT* (Bruker, 2007 ▸), *SHELXS* (Sheldrick, 2008 ▸), *SHELXL2016/6* (Sheldrick, 2015 ▸), *ORTEP-3 for Windows* (Farrugia, 2012 ▸) and *PLATON* (Spek, 2020 ▸).

## supplementary crystallographic information

### Crystal data

**Table:**

C_16_H_12_F_5_N_3_O	*F*(000) = 1456
*M**_r_* = 357.29	*D*_x_ = 1.407 Mg m^−^^3^
Monoclinic, *C*2/*c*	Mo *K*α radiation, λ = 0.71073 Å
*a* = 19.0048 (6) Å	Cell parameters from 9937 reflections
*b* = 11.5216 (4) Å	θ = 3.0–30.5°
*c* = 17.2227 (6) Å	µ = 0.13 mm^−^^1^
β = 116.526 (1)°	*T* = 296 K
*V* = 3374.2 (2) Å^3^	Prism, colourless
*Z* = 8	0.86 × 0.76 × 0.32 mm

### Data collection

**Table:**

Bruker APEXII CCD diffractometer	3075 reflections with *I* > 2σ(*I*)
φ and ω scans	*R*_int_ = 0.025
Absorption correction: multi-scan (SADABS; Krause *et al.*, 2015)	θ_max_ = 27.0°, θ_min_ = 3.0°
*T*_min_ = 0.666, *T*_max_ = 0.746	*h* = −24→24
20072 measured reflections	*k* = −14→14
3619 independent reflections	*l* = −21→21

### Refinement

**Table:**

Refinement on *F*^2^	Primary atom site location: structure-invariant direct methods
Least-squares matrix: full	Hydrogen site location: mixed
*R*[*F*^2^ > 2σ(*F*^2^)] = 0.044	H atoms treated by a mixture of independent and constrained refinement
*wR*(*F*^2^) = 0.129	*w* = 1/[σ^2^(*F*_o_^2^) + (0.066*P*)^2^ + 1.0984*P*] where *P* = (*F*_o_^2^ + 2*F*_c_^2^)/3
*S* = 1.08	(Δ/σ)_max_ < 0.001
3619 reflections	Δρ_max_ = 0.27 e Å^−^^3^
232 parameters	Δρ_min_ = −0.16 e Å^−^^3^
1 restraint	

### Special details

**Table:**

Geometry. All esds (except the esd in the dihedral angle between two l.s. planes) are estimated using the full covariance matrix. The cell esds are taken into account individually in the estimation of esds in distances, angles and torsion angles; correlations between esds in cell parameters are only used when they are defined by crystal symmetry. An approximate (isotropic) treatment of cell esds is used for estimating esds involving l.s. planes.

### Fractional atomic coordinates and isotropic or equivalent isotropic displacement parameters (Å^2^)

**Table:**

	*x*	*y*	*z*	*U*_iso_*/*U*_eq_	
C1	0.57129 (9)	0.67608 (14)	0.56090 (9)	0.0560 (4)	
C2	0.62879 (10)	0.6234 (2)	0.54530 (11)	0.0709 (5)	
C3	0.64867 (9)	0.5107 (2)	0.57013 (12)	0.0741 (6)	
C4	0.61080 (9)	0.45216 (16)	0.60894 (11)	0.0675 (5)	
C5	0.55429 (8)	0.50569 (13)	0.62548 (9)	0.0526 (3)	
C6	0.53295 (7)	0.62075 (12)	0.60236 (8)	0.0441 (3)	
C7	0.47580 (7)	0.68280 (11)	0.62380 (7)	0.0396 (3)	
C8	0.49138 (7)	0.81069 (11)	0.64576 (8)	0.0405 (3)	
C9	0.44817 (14)	1.00857 (16)	0.61948 (15)	0.0898 (6)	
H9A	0.503442	1.022817	0.652886	0.135*	
H9B	0.420993	1.030799	0.652629	0.135*	
H9C	0.428581	1.053157	0.566896	0.135*	
C10	0.36406 (10)	0.85441 (17)	0.52042 (11)	0.0755 (5)	
H10A	0.363739	0.772275	0.510888	0.113*	
H10B	0.363100	0.895011	0.471289	0.113*	
H10C	0.318605	0.875319	0.527986	0.113*	
C11	0.31918 (7)	0.60410 (11)	0.67138 (7)	0.0409 (3)	
C12	0.27412 (8)	0.65818 (13)	0.70541 (9)	0.0505 (3)	
H12	0.281692	0.736455	0.720011	0.061*	
C13	0.21793 (9)	0.59576 (16)	0.71764 (11)	0.0635 (4)	
H13	0.187876	0.632183	0.740795	0.076*	
C14	0.20605 (9)	0.48080 (17)	0.69601 (11)	0.0698 (5)	
H14	0.167784	0.439171	0.703856	0.084*	
C15	0.25111 (10)	0.42742 (15)	0.66262 (11)	0.0678 (4)	
H15	0.243095	0.349161	0.648076	0.081*	
C16	0.30814 (9)	0.48750 (13)	0.65015 (9)	0.0530 (3)	
H16	0.338580	0.450219	0.627863	0.064*	
N1	0.43528 (7)	0.88584 (11)	0.59834 (8)	0.0539 (3)	
N2	0.42258 (6)	0.62135 (9)	0.63136 (6)	0.0417 (3)	
N3	0.37406 (6)	0.67219 (10)	0.65833 (7)	0.0450 (3)	
O1	0.55445 (5)	0.84009 (8)	0.70599 (6)	0.0477 (2)	
F1	0.55220 (7)	0.78590 (9)	0.53338 (7)	0.0777 (3)	
F2	0.66439 (8)	0.68233 (13)	0.50601 (9)	0.1088 (5)	
F3	0.70507 (6)	0.45858 (14)	0.55668 (9)	0.1114 (5)	
F4	0.62894 (7)	0.34098 (10)	0.63199 (9)	0.0994 (4)	
F5	0.52239 (6)	0.44377 (8)	0.66683 (7)	0.0724 (3)	
H3N	0.3875 (9)	0.7382 (12)	0.6861 (10)	0.054 (4)*	

### Atomic displacement parameters (Å^2^)

**Table:**

	*U*^11^	*U*^22^	*U*^33^	*U*^12^	*U*^13^	*U*^23^
C1	0.0493 (8)	0.0732 (10)	0.0498 (7)	−0.0048 (7)	0.0261 (6)	−0.0096 (7)
C2	0.0543 (9)	0.1071 (15)	0.0627 (9)	−0.0149 (9)	0.0364 (8)	−0.0285 (9)
C3	0.0426 (8)	0.1094 (15)	0.0668 (10)	0.0056 (9)	0.0212 (7)	−0.0389 (10)
C4	0.0506 (8)	0.0744 (11)	0.0600 (9)	0.0171 (7)	0.0089 (7)	−0.0206 (8)
C5	0.0418 (7)	0.0594 (8)	0.0477 (7)	0.0051 (6)	0.0121 (6)	−0.0074 (6)
C6	0.0334 (6)	0.0566 (7)	0.0379 (6)	0.0006 (5)	0.0120 (5)	−0.0064 (5)
C7	0.0327 (6)	0.0469 (7)	0.0363 (6)	0.0012 (5)	0.0127 (4)	0.0019 (5)
C8	0.0371 (6)	0.0464 (7)	0.0414 (6)	0.0012 (5)	0.0205 (5)	0.0072 (5)
C9	0.1062 (16)	0.0507 (9)	0.0990 (15)	0.0159 (10)	0.0337 (12)	0.0198 (9)
C10	0.0611 (10)	0.0881 (12)	0.0561 (9)	0.0207 (9)	0.0070 (7)	0.0169 (8)
C11	0.0315 (6)	0.0488 (7)	0.0371 (6)	−0.0040 (5)	0.0106 (5)	0.0028 (5)
C12	0.0396 (6)	0.0577 (8)	0.0542 (7)	0.0002 (6)	0.0211 (6)	0.0032 (6)
C13	0.0446 (7)	0.0856 (11)	0.0660 (9)	0.0030 (7)	0.0299 (7)	0.0159 (8)
C14	0.0475 (8)	0.0849 (12)	0.0746 (10)	−0.0139 (8)	0.0250 (7)	0.0232 (9)
C15	0.0670 (10)	0.0552 (9)	0.0730 (10)	−0.0188 (7)	0.0239 (8)	0.0062 (7)
C16	0.0527 (8)	0.0511 (8)	0.0533 (7)	−0.0072 (6)	0.0220 (6)	−0.0016 (6)
N1	0.0531 (7)	0.0507 (7)	0.0530 (7)	0.0100 (5)	0.0193 (5)	0.0129 (5)
N2	0.0340 (5)	0.0477 (6)	0.0415 (5)	−0.0013 (4)	0.0152 (4)	−0.0029 (4)
N3	0.0406 (6)	0.0448 (6)	0.0540 (6)	−0.0072 (4)	0.0250 (5)	−0.0087 (5)
O1	0.0402 (5)	0.0487 (5)	0.0521 (5)	−0.0068 (4)	0.0186 (4)	0.0037 (4)
F1	0.0964 (8)	0.0791 (7)	0.0827 (7)	−0.0027 (6)	0.0624 (6)	0.0109 (5)
F2	0.1025 (9)	0.1503 (12)	0.1183 (10)	−0.0331 (8)	0.0893 (8)	−0.0381 (9)
F3	0.0624 (7)	0.1649 (13)	0.1112 (9)	0.0196 (7)	0.0426 (6)	−0.0552 (9)
F4	0.0920 (8)	0.0804 (8)	0.1078 (9)	0.0392 (6)	0.0285 (7)	−0.0140 (6)
F5	0.0759 (6)	0.0556 (5)	0.0884 (7)	0.0128 (4)	0.0389 (5)	0.0134 (5)

### Geometric parameters (Å, º)

**Table:**

C1—F1	1.3430 (19)	C9—H9C	0.9600
C1—C2	1.377 (2)	C10—N1	1.463 (2)
C1—C6	1.383 (2)	C10—H10A	0.9600
C2—F2	1.336 (2)	C10—H10B	0.9600
C2—C3	1.367 (3)	C10—H10C	0.9600
C3—F3	1.3359 (18)	C11—C12	1.3832 (19)
C3—C4	1.360 (3)	C11—C16	1.3835 (19)
C4—F4	1.340 (2)	C11—N3	1.4011 (16)
C4—C5	1.374 (2)	C12—C13	1.380 (2)
C5—F5	1.3301 (18)	C12—H12	0.9300
C5—C6	1.392 (2)	C13—C14	1.367 (3)
C6—C7	1.4783 (17)	C13—H13	0.9300
C7—N2	1.2880 (16)	C14—C15	1.372 (3)
C7—C8	1.5172 (18)	C14—H14	0.9300
C8—O1	1.2317 (15)	C15—C16	1.381 (2)
C8—N1	1.3325 (16)	C15—H15	0.9300
C9—N1	1.453 (2)	C16—H16	0.9300
C9—H9A	0.9600	N2—N3	1.3385 (14)
C9—H9B	0.9600	N3—H3N	0.873 (13)
			
F1—C1—C2	117.32 (15)	N1—C10—H10B	109.5
F1—C1—C6	119.67 (13)	H10A—C10—H10B	109.5
C2—C1—C6	123.01 (17)	N1—C10—H10C	109.5
F2—C2—C3	120.62 (16)	H10A—C10—H10C	109.5
F2—C2—C1	120.0 (2)	H10B—C10—H10C	109.5
C3—C2—C1	119.34 (17)	C12—C11—C16	119.99 (12)
F3—C3—C4	120.3 (2)	C12—C11—N3	117.50 (12)
F3—C3—C2	120.2 (2)	C16—C11—N3	122.50 (12)
C4—C3—C2	119.47 (14)	C13—C12—C11	119.85 (14)
F4—C4—C3	119.80 (16)	C13—C12—H12	120.1
F4—C4—C5	119.29 (18)	C11—C12—H12	120.1
C3—C4—C5	120.90 (17)	C14—C13—C12	120.56 (16)
F5—C5—C4	117.09 (14)	C14—C13—H13	119.7
F5—C5—C6	121.33 (12)	C12—C13—H13	119.7
C4—C5—C6	121.55 (15)	C13—C14—C15	119.37 (14)
C1—C6—C5	115.69 (13)	C13—C14—H14	120.3
C1—C6—C7	121.43 (13)	C15—C14—H14	120.3
C5—C6—C7	122.82 (12)	C14—C15—C16	121.37 (16)
N2—C7—C6	117.21 (12)	C14—C15—H15	119.3
N2—C7—C8	125.67 (11)	C16—C15—H15	119.3
C6—C7—C8	116.65 (10)	C15—C16—C11	118.85 (15)
O1—C8—N1	123.28 (12)	C15—C16—H16	120.6
O1—C8—C7	119.03 (11)	C11—C16—H16	120.6
N1—C8—C7	117.68 (11)	C8—N1—C9	118.65 (14)
N1—C9—H9A	109.5	C8—N1—C10	124.09 (13)
N1—C9—H9B	109.5	C9—N1—C10	116.99 (14)
H9A—C9—H9B	109.5	C7—N2—N3	119.24 (11)
N1—C9—H9C	109.5	N2—N3—C11	119.22 (11)
H9A—C9—H9C	109.5	N2—N3—H3N	119.5 (10)
H9B—C9—H9C	109.5	C11—N3—H3N	117.2 (11)
N1—C10—H10A	109.5		
			
F1—C1—C2—F2	1.5 (2)	C5—C6—C7—N2	−28.19 (17)
C6—C1—C2—F2	−179.03 (14)	C1—C6—C7—C8	−32.65 (16)
F1—C1—C2—C3	−178.29 (14)	C5—C6—C7—C8	144.48 (12)
C6—C1—C2—C3	1.2 (2)	N2—C7—C8—O1	114.61 (14)
F2—C2—C3—F3	1.2 (2)	C6—C7—C8—O1	−57.36 (15)
C1—C2—C3—F3	−179.06 (14)	N2—C7—C8—N1	−65.23 (16)
F2—C2—C3—C4	−179.10 (15)	C6—C7—C8—N1	122.80 (12)
C1—C2—C3—C4	0.7 (2)	C16—C11—C12—C13	−0.4 (2)
F3—C3—C4—F4	−1.5 (2)	N3—C11—C12—C13	178.48 (12)
C2—C3—C4—F4	178.76 (14)	C11—C12—C13—C14	−0.3 (2)
F3—C3—C4—C5	178.08 (13)	C12—C13—C14—C15	0.6 (2)
C2—C3—C4—C5	−1.7 (2)	C13—C14—C15—C16	−0.2 (3)
F4—C4—C5—F5	2.3 (2)	C14—C15—C16—C11	−0.5 (2)
C3—C4—C5—F5	−177.31 (13)	C12—C11—C16—C15	0.7 (2)
F4—C4—C5—C6	−179.60 (13)	N3—C11—C16—C15	−178.03 (13)
C3—C4—C5—C6	0.8 (2)	O1—C8—N1—C9	−1.2 (2)
F1—C1—C6—C5	177.51 (12)	C7—C8—N1—C9	178.61 (14)
C2—C1—C6—C5	−2.0 (2)	O1—C8—N1—C10	172.55 (14)
F1—C1—C6—C7	−5.2 (2)	C7—C8—N1—C10	−7.6 (2)
C2—C1—C6—C7	175.37 (13)	C6—C7—N2—N3	174.02 (10)
F5—C5—C6—C1	179.00 (12)	C8—C7—N2—N3	2.09 (18)
C4—C5—C6—C1	0.95 (19)	C7—N2—N3—C11	−176.33 (11)
F5—C5—C6—C7	1.72 (19)	C12—C11—N3—N2	175.29 (11)
C4—C5—C6—C7	−176.33 (12)	C16—C11—N3—N2	−5.90 (18)
C1—C6—C7—N2	154.68 (12)		

### Hydrogen-bond geometry (Å, º)

*Cg*2 is the centroid of the C11–C16 ring.

**Table:**

*D*—H···*A*	*D*—H	H···*A*	*D*···*A*	*D*—H···*A*
N3—H3*N*···O1^i^	0.87 (1)	2.05 (2)	2.8658 (15)	154 (1)
C9—H9*A*···O1	0.96	2.33	2.717 (2)	103
C10—H10*A*···N2	0.96	2.55	3.194 (2)	124
C14—H14···O1^ii^	0.93	2.45	3.377 (2)	172
C10—H10*B*···*Cg*2^iii^	0.96	2.77	3.4685 (19)	130

Symmetry codes: (i) −*x*+1, *y*, −*z*+3/2; (ii) *x*−1/2, *y*−1/2, *z*; (iii) −*x*+1/2, −*y*+3/2, −*z*+1.
