# Supplementary figures and images for: Crystal structure and Hirshfeld surface analysis of (2Z)-N,N-dimethyl-2-(penta­fluoro­phen­yl)-2-(2-phenyl­hydrazin-1-yl­idene)acetamide
Source: Acta Crystallogr E Crystallogr Commun. 2021 Jul 23;77(Pt 8):829–33. doi: 10.1107/S2056989021007349 (PMC8340965; doi:10.1107/S2056989021007349)

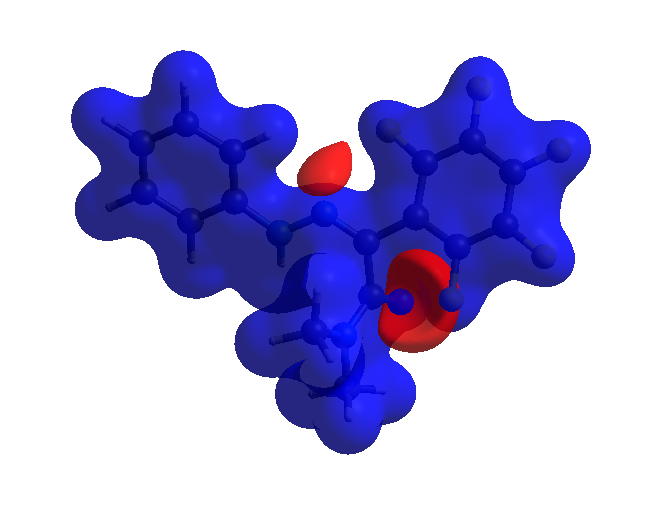


Supplementary figure 1: Hirshfeld surface mapped over electrostatic potential

Supplement: Supplementary file 3 [file e-77-00829-sup3.docx]
